# ﻿*Caelospermum* versus *Coelospermum* in Rubiaceae (Gentianales): their etymologies explained

**DOI:** 10.3897/phytokeys.252.136744

**Published:** 2025-02-14

**Authors:** Brecht Verstraete, Elmar Robbrecht

**Affiliations:** 1 Meise Botanic Garden, Meise, Belgium Meise Botanic Garden Meise Belgium

**Keywords:** Carl Ludwig Blume, etymology, Greek, Latin, nomenclature, orthography, Rubiaceae

## Abstract

*Caelospermum* is the original spelling of the generic name, as it appeared in the protologue, but in literature and online databases, the variant spelling *Coelospermum* is often used. However, the confusion about the spelling is caused by a misinterpretation of the etymology. Here, we demonstrate that the original spelling by Blume should be retained.

## ﻿*Caelospermum* versus *Coelospermum*

*Caelospermum* Blume is a genus within the Rubiaceae family that is found in Southeast Asia and Oceania and currently consists of 12 species (POWO 2025). The genus is included in the tribe Morindeae, subfamily Rubioideae (Razafimandimbison and Rydin 2024). The circumscription of the genus has been controversial but a broadly delimited *Caelospermum* can be characterised by the mostly lianescent habit, corolla tubes with narrow longitudinal slits alternating with the lobes, ovaries simple or 2–20(–30)-connate, primarily bilocular with biovular locules, later becoming 4-locular by the development of secondary septa, drupaceous fruits, concavo-convex to planoconvex pyrenes with a narrow slit in the lower part, and flattened seeds with a narrow basal wing (Johansson 1988). Razafimandimbison et al. (2009) confirmed Johansson’s concept of *Caelospermum* with molecular phylogenetic methods, although enlarging it by treating the Australian genus *Pogonolobus* F.Muell. as a synonym.

**Figure 1.**
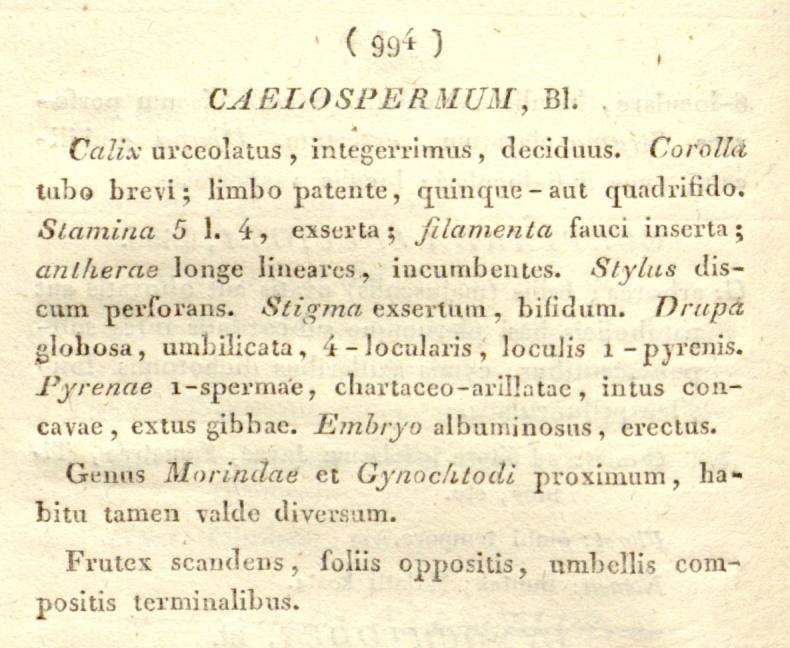
The original publication of the generic name *Caelospermum* Blume (from Blume 1826).

The generic name was originally spelled as *Caelospermum* in the protologue (Blume 1826: 994; Fig. 1). The genus was mentioned to be closely related to *Morinda* L. and *Gynochtodes* Blume and a single species (i.e. the type species), *Caelospermumscandens* Blume, was described from Nusa Kambangan island in Indonesia. There was no explanation of the etymology of the generic name, but it seems evident that it is derived from Latin “caelum” (= heaven, sky) and “sperma” (= seed). Although Blume mentioned some characters of the pyrenes (Fig. 1), this etymology did not seem to make sense.

Shortly after, the spelling of the generic name was changed by other taxonomists. Both de Candolle (1830: 468) and Richard (1834: 209) corrected the name to *Coelospermum*, however, without commenting on the reason. Hooker (1873: 119) also used *Coelospermum* but he mentioned *Caelospermum* between brackets. Throughout literature, both spelling variants have been used, although it seems that *Coelospermum* is more prevalent, especially in recent molecular phylogenetic studies (e.g. Razafimandimbison et al. 2008, 2009). The monographer of the genus first used *Coelospermum* (Johansson 1987). In his revision of the genus, however, he used the original spelling *Caelospermum* and listed the name *Coelospermum* in synonymy (Johansson 1988), without explaining why he made this change. Other authors also used both variants in different publications (e.g. Halford and Ford 2004 vs Halford and Ford 2009). Mouly and Fleurot (2021) used the spelling *Coelospermum* but explicitly mentioned *Caelospermum* as an orthographical variant. The origin of the change in spelling is found in the interpretation of the etymology. Subsequent authors interpreted the name as derived from Greek “koilos” (= concave, hollow), not Latin “caelum” (= heaven, sky). Since Blume (1826)’s description included the phrase “Pyrenae ... intus concavae ...”, it was interpreted that Blume meant “hollow-seeded” (IPNI 2025).

Besides in literature, the names are also entered in online databases about nomenclature and taxonomy. The International Plant Names Index (IPNI 2025) lists both *Caelospermum* and *Coelospermum* but both names have two entries and there is confusion about the correct spelling. In the nomenclatural notes of *Caelospermum* (https://www.ipni.org/n/34272-1), it says to use *Coelospermum*. However, the entry *Caelospermum* (https://www.ipni.org/n/116925-3) remarks that there is an orthographical variant *Coelospermum*. The entry *Coelospermum* (https://www.ipni.org/n/327850-2) has a remark in which IPNI chooses to correct *Caelospermum* to *Coelospermum*. The entry *Coelospermum* (https://www.ipni.org/n/85291-3) says the opposite because the remark says that *Coelospermum* is the orthographical variant. Tropicos (Tropicos.org 2025) is clearer about the spelling: *Coelospermum* (https://www.tropicos.org/name/40037911) is considered to be the orthographical variant of *Caelospermum* (https://www.tropicos.org/name/40034119). The World Flora Online (WFO 2025) lists *Caelospermum* (https://www.worldfloraonline.org/taxon/wfo-4000008840) and several subordinate taxa, but all those names are annotated as unchecked names. The status of *Coelospermum* (https://www.worldfloraonline.org/taxon/wfo-4000005905) and its species are indicated as accepted names. The Plants of the World Online (POWO 2025 only lists *Coelospermum* (https://powo.science.kew.org/taxon/urn:lsid:ipni.org:names:327850-2).

The interchangeability of cael- (from “caelum”) and coel- (from “koilos”) is also noted for other generic names in angiosperms. Hasskarl (1855: 139) published the name *Koilodepas* Hassk. (Euphorbiaceae), which is derived from Greek “koilos” (= concave) and “depas” (= beaker) and refers to the cupulate calyx. He later Latinised the name to *Coelodepas* Hassk. (Hasskarl, 1857). However, Bentham and Hooker (1880: 313) published the name as *Caelodepas* Benth. & Hook.f., with reference to *Coelodepas* Hassk. and *Koilodepas* Hassk. Other examples are *Caelebogyne* J.Sm. / *Coelebogyne* J.Sm., *Caelestina* Cass. / *Coelestina* Cass., *Caelia* G.Don. / *Coelia* Lindl., *Caelocline* Auct. ex Steud. / *Coelocline* A.DC., *Caeloglossum* Steud. / *Coeloglossum* Lindl., and *Caelogyne* Wall. ex Steud. / *Coelogyne* Lindl. (Steudel 1840: 247; IPNI 2025). In almost all cases, the variant coel- is preferred (POWO 2025; WFO 2025). *Caelestium* Yurtseva & Mavrodiev (Polygonaceae) has no variant because the etymology is explicitly stated in the protologue: “… from “caelestis” (= heavenly, divinus)” (Yurtseva and Mavrodiev 2019: 73).

**Figure 2.**
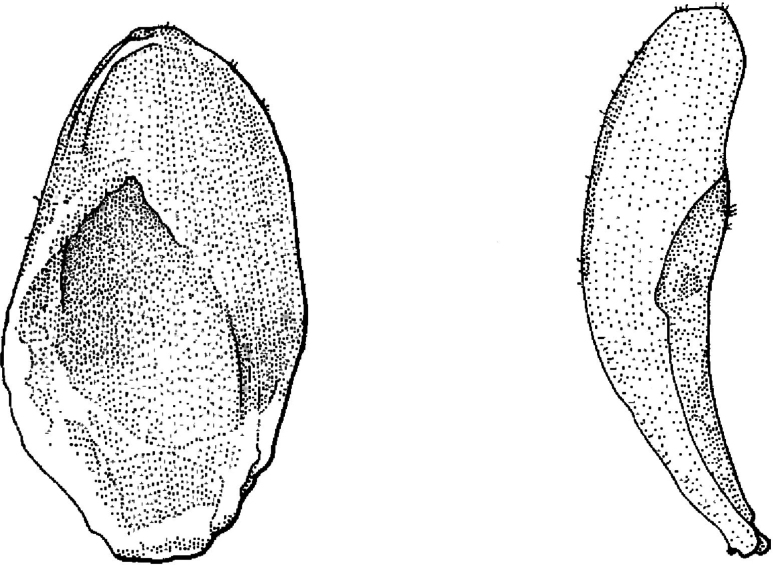
The concave pyrene with a basal marginal groove of *Caelospermumpurpureum* Halford & A.J.Ford in adaxial (left) and lateral view (right) (reproduced from Halford and Ford 2009: fig. 1J, K with permission from the editor).

Regarding *Caelospermum*, the correction of Blume’s spelling of the name is the result of a misinterpretation of the etymology. *Caelospermum* can indeed be derived from Latin “caelum” but it should be translated as “vault of heaven” (Lewis and Short 1891). In ancient cosmology, the celestial vault is portrayed as a vast solid dome arching above the Earth. The name would then refer to arched pyrenes (Fig. 2), which corresponds well with the characters in the protologue (i.e. “Pyrenae … intus concavae, extus gibbae”) and in Johansson (1988)’s revision (i.e. “endocarp … concavo-convex to planoconvex”). The interpretation that the name would be derived from Greek “koilos” is conceivable but not necessarily correct.

In Rubiaceae, the origins of the generic names by Blume are not always obvious (e.g. *Metabolos* Blume; Bremekamp 1952: 30) and Blume never explained their etymologies in the protologues, except for eponyms. We will therefore never be able to definitively ascertain what Blume actually intended. Ultimately, both cael- and coel- can refer to a concave feature, and can therefore be used in that sense. However, their etymologies are different, so they should not be considered as orthographical variants. In that case, the original spelling of the name should be retained (ICN Art. 60.1; Turland 2019). In conclusion, the original spelling *Caelospermum* Blume is correct and should be used.

## ﻿Additional information

### Author ORCIDs

Brecht Verstraete https://orcid.org/0000-0001-9332-7798

Elmar Robbrecht https://orcid.org/0000-0002-3811-595X

### Data availability

All of the data that support the findings of this study are available in the main text.
